# Contrasting Granite Metallogeny through the Zircon Record: A Case Study from Myanmar

**DOI:** 10.1038/s41598-017-00832-2

**Published:** 2017-04-07

**Authors:** Nicholas J. Gardiner, Chris J. Hawkesworth, Laurence J. Robb, Martin J. Whitehouse, Nick M. W. Roberts, Christopher L. Kirkland, Noreen J. Evans

**Affiliations:** 1 Department of Earth Sciences, University of Oxford, South Parks Road, Oxford, OX1 3AN UK; 2 Centre for Exploration Targeting – Curtin Node, Department of Applied Geology, Western Australian School of Mines, Curtin University, Perth, WA 6102 Australia; 3 School of Earth Sciences, University of Bristol, Bristol, BS8 1RJ UK; 4 Department of Geological Sciences, Swedish Museum of Natural History, Box 50007, Stockholm, Sweden; 5 NERC Isotope Geosciences Laboratory, British Geological Survey, Keyworth, NG12 5GG UK; 6 John De Laeter Centre, TIGeR, Department of Applied Geology, Curtin University, Perth, WA 6102 Australia

## Abstract

Granitoid-hosted mineral deposits are major global sources of a number of economically important metals. The fundamental controls on magma metal fertility are tectonic setting, the nature of source rocks, and magma differentiation. A clearer understanding of these petrogenetic processes has been forged through the accessory mineral zircon, which has considerable potential in metallogenic studies. We present an integrated zircon isotope (U-Pb, Lu-Hf, O) and trace element dataset from the paired Cu-Au (copper) and Sn-W (tin) magmatic belts in Myanmar. Copper arc zircons have juvenile εHf (+7.6 to +11.5) and mantle-like δ^18^O (5.2–5.5‰), whereas tin belt zircons have low εHf (−7 to −13) and heavier δ^18^O (6.2–7.7‰). Variations in zircon Hf and U/Yb reaffirm that tin belt magmas contain greater crustal contributions than copper arc rocks. Links between whole-rock Rb/Sr and zircon Eu/Eu* highlight that the latter can monitor magma fractionation in these systems. Zircon Ce/Ce* and Eu/Eu* are sensitive to redox and fractionation respectively, and here are used to evaluate zircon sensitivity to the metallogenic affinity of their host rock. Critical contents of Sn in granitic magmas, which may be required for the development of economic tin deposits, are marked by zircon Eu/Eu* values of ca. ≤0.08.

## Introduction

Granitoid-hosted mineral deposits are global sources for Cu, Mo, Sn, W, Au, U, Ta and Nb. Magmatic genesis and evolution exerts a fundamental control on the propensity of granitoids to be metal-fertile^1^. A revolution in our understanding of granite petrogenesis has been forged through mineral-based tools, principally the accessory mineral zircon^2, 3^. Zircons are chemically robust, they can be precisely dated, they are reliable monitors of the evolution of their parental magmas, and they survive a range of geological processes that can impinge upon the reliability of whole-rock data^4^. Advances in micro-analysis have resulted in the routine *in-situ* measurement of key geochemical and isotopic traits in zircons formed within a range of tectonic settings^5^, and these techniques are increasingly applied to metallogenic problems. Over the past decade there has been increased interest in the use of accessory minerals as so-called pathfinders to prospective areas of granite-hosted mineralization, largely focusing on Cu-Au(-Mo) porphyry deposits, and the role of zircon rare earth elements as oxy-barometers^6–8^.

Magmatic source, redox state, and the degree of magma fractionation, are petrogenetic factors that control the development of metal-fertile magmas leading to magmatic-hydrothermal mineral deposits^9^. Identification of traits in zircon sensitive to these factors may potentially be developed into exploration tools, however what is required is a better understanding of how the traits respond to different types of granite metallogeny. One approach is to contrast zircon chemistry in granite suites that host distinct types of mineral deposits. The paired magmatic belts of Myanmar offer an ideal case study since they host contrasting metallogeny: vein-and-pegmatite Sn-W and porphyry-type Cu-Au.

We present new zircon data from the Myanmar belts, focusing on geochemical and isotopic traits that fingerprint source, redox, and the degree of magma differentiation. Evaluating their sensitivity to these controls, we consider the extent to which magmatic, and by implication detrital zircons offer new ways to constrain the petrogenetic factors that may promote the formation of mineral deposits. The zircon Eu anomaly records magma fractionation, and using the example of Sn mineralization, an incompatible metal whose concentration is highly sensitive to the degree of fractionation, we show how zircon chemistry can be used to establish thresholds that may distinguish barren from fertile granites.

## Background

### Granitoid-hosted Mineral Deposits

Granitoid-hosted mineral deposits are typically subdivided into “Cu-Au- (±Mo)” and “Sn-W” categories, which are associated with oxidized intermediate magmas, and reduced more fractionated felsic magmas, respectively^10^. Ore-forming magmas have gross chemical and mineralogical similarities to barren magmas. Investigations into the controls on magma metal endowment and sequestration therefore focus on subtle petrogenetic differences. Empirical observations have invoked the role of source and fractionation as key factors controlling granite metallogeny^9^.

Source, the inherited properties of the lithospheric domain from which the melt was extracted, determines the initial metal contents of the magmas. Source also imposes a geochemical heredity onto the evolving magma system, most crucially oxygen fugacity, or redox^11^. Redox exerts a genetic control on the metallogenetic potential for both Cu-Au and Sn-W mineralization^1, 9, 12^, controlling sulphur speciation, with significant implications for the availability of both chalcophile and siderophile metals (Cu, Mo, Ni, Au) within the residual melt. Redox also controls metal solubilities, for example Sn speciation (Sn^2+^ or Sn^4+^) determines whether Sn substitutes into crystallizing phases in oxidized melts, or remains residually enriched in more reduced magmas.

The role of fractionation and magma differentiation are processes that may enhance the concentration of metals during progressive magma crystallization. Incompatible metals (e.g., Sn, U, Ta, Nb) are residually concentrated in the melt, and may be scavenged by hydrothermal fluids ultimately to form ore minerals (such as cassiterite) during the processes of mineralization.

### Geology

**Figure 1:**
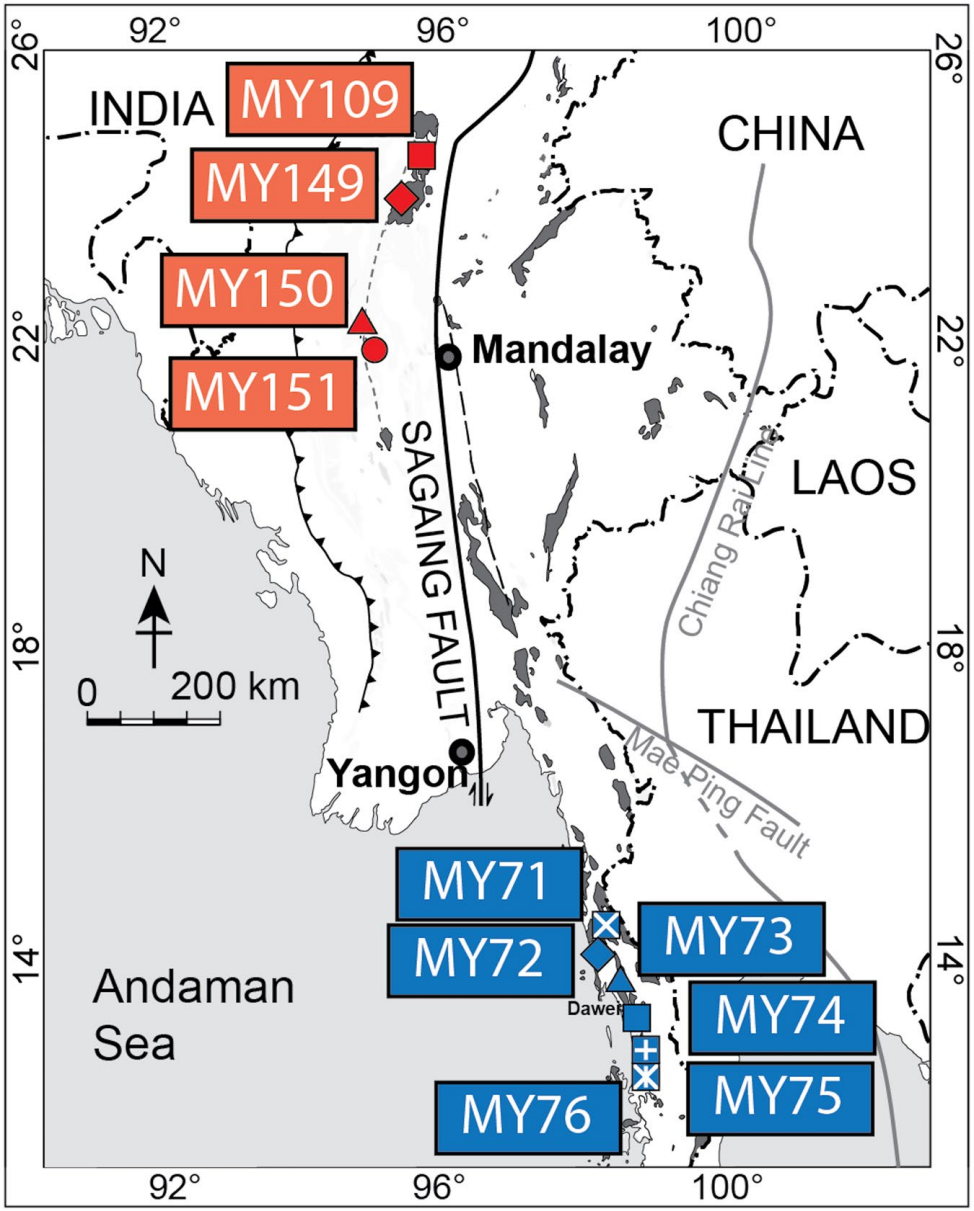
Outline map of Myanmar with sample localities of the tin belt (blue) and copper belt (red). The original juxtaposition of the two belts has been offset by dextral movement on the north-trending Sagaing Fault. Map made with Natural Earth Data http://www.naturalearthdata.com/.

The paired magmatic belts of Myanmar are interpreted to have formed on the subducting margin of Neo-Tethys during the Late Mesozoic-Early Cenozoic^13^. A western magmatic arc comprises I-type granites hosting Cu-Au porphyry mineralization (‘copper arc’). An eastern belt of S-type granites hosts significant Sn-W mineralization (‘tin belt’). Their geodynamic setting and metallogenic affinity is similar to the metallogenic belts of the Central Andes^14^, although Myanmar experienced a simpler and shorter geological history in a more constrained geographic area. Samples of granitoids were taken from ten localities adjacent to known mining areas, and they are all un-mineralized rocks that represent the metallogenic affinities of the two belts (Fig. 1; Table 1).

**Table 1:** Sample locations and a summary of key isotope and geochemical metrics as discussed in the text. *U-Pb age from Gardiner *et al*. (2016)^13^.

Belt	Sample	Locality	Description	N/E	U-Pb age (2σ)	εHf (2σ)	δ^18^O (2σ)	Ce/Ce* (1σ)	Eu/Eu* (1σ)
Sn	MY71	Dawei	bt + pl + kfs granite	14°16′32′′′N/98°15′37′′E	75.6 ± 8.8	−10.4 ± 1.1	6.3 ± 0.3	4.2 ± 2.9	0.03 ± 0.0
	MY72	Dawei	bt + pl + kfs granite	14°08′15′′N/98°07′06′′E	64.1 ± 1.6	−7.0 ± 1.0	6.6 ± 0.3	16.9 ± 392	0.21 ± 0.1
	MY73		bt + pl + kfs granite	13°40′12′′ N/98°23′00′′E	58.5 ± 0.5	−13 ± 1.2	7.1 ± 0.3	42.9 ± 383	0.24 ± 0.1
	MY74		bt + pl + kfs granite	13°34′05′′N/ 98°25′13′′E	58.7 ± 0.6	−9.4 ± 1.1	7.2 ± 0.3	13.6 ± 41	0.20 ± 0.0
	MY75	Myeik	bt + pl + kfs granite	12°41′31′′N/ 98°44′12′′E	72.1 ± 1.3	−10.3 ± 1.4	7.7 ± 0.3	18.1 ± 71	0.07 ± 0.0
	MY76	Myeik	bt + pl + kfs granite	12°29′36′′N/ 98°41′55′′E	75.3 ± 7.7*	−10.2 ± 3.4	6.2 ± 0.3	0.82 ± 3.7	0.04 ± 0.1
Cu	MY109	Banmauk	hbl + bt + pl granodiorite (sulphides)	24°24′46′′N/95°45′35′′E	102.1 ± 0.95	11.5 ± 1.2	5.2 ± 0.2	46.5 ± 28	0.24 ± 0.0
	MY149	Wuntho	hbl + bt + pl granodiorite	24°00′12′′N/95°27′56′′E	98.1 ± 0.47	7.6 ± 0.9	5.4 ± 0.3	349.6 ± 2743	0.21 ± 0.0
	MY150	Monywa	hbl + bt + pl granodiorite	22°10′30′′N/94°58′05′′E	99.8 ± 1.3	7.3 ± 1.4	5.4 ± 0.4	105.6 ± 112	0.19 ± 0.0
	MY151	Salyingyi	hbl + pl ( + bt) diorite	21°56′49′′N/95°05′32′′E	98.3 ± 1.0	7.4 ± 1.7	5.5 ± 0.4	182.2 ± 120	0.22 ± 0.0

### Approach

The mineral zircon provides an exceptional record of magmatic evolution^15^. Modern *in-situ* analytical techniques (SIMS, LA-ICP-(MC)-MS) allow the precise measurement of U-Pb, Lu-Hf, and O isotopes, and trace elements in zircons, and which arguably show greater fidelity to magmatic processes than whole-rock analysis. We apply these techniques to zircons from the Myanmar samples.

Separated zircon grains were dated through U-Pb geochronology, and their oxygen isotope compositions determined using ion microprobe analysis. O isotopes (^18^O/^16^O ratio, expressed as δ^18^O V-SMOW) are sensitive to source: values above a mantle signature (δ^18^O = 5.3‰) fingerprint a contribution from ^18^O-enriched material which has experienced Earth surface processes, pointing to a greater supercrustal component in the melt. Zircon ^176^Hf/^177^Hf isotope ratios were determined using LA-ICP-MC-MS, targeting the same domains that yielded the U-Pb and O isotope results. Hf isotopic signatures, expressed as εHf, provide a measure of magma source composition, indicating the extent to which the melt was either juvenile, i.e. close to a mantle value and enriched in radiogenic ^176^Hf (higher εHf); or derived from an evolved, typically crustal source (i.e. with lower εHf). Zircon trace element analyses, which may inform on a range of geochemical conditions and geodynamic settings, were undertaken using LA-ICP-MS. The net result is an integrated dataset of coupled U-Pb, Lu-Hf, O isotopes, and trace element data, for zircon domains, plus whole rock major and trace element data, across the range of samples from both belts (full methodology and results in the supplementary information and data tables).

## Discussion

### Constraining Magmatic Source

**Figure 2:**
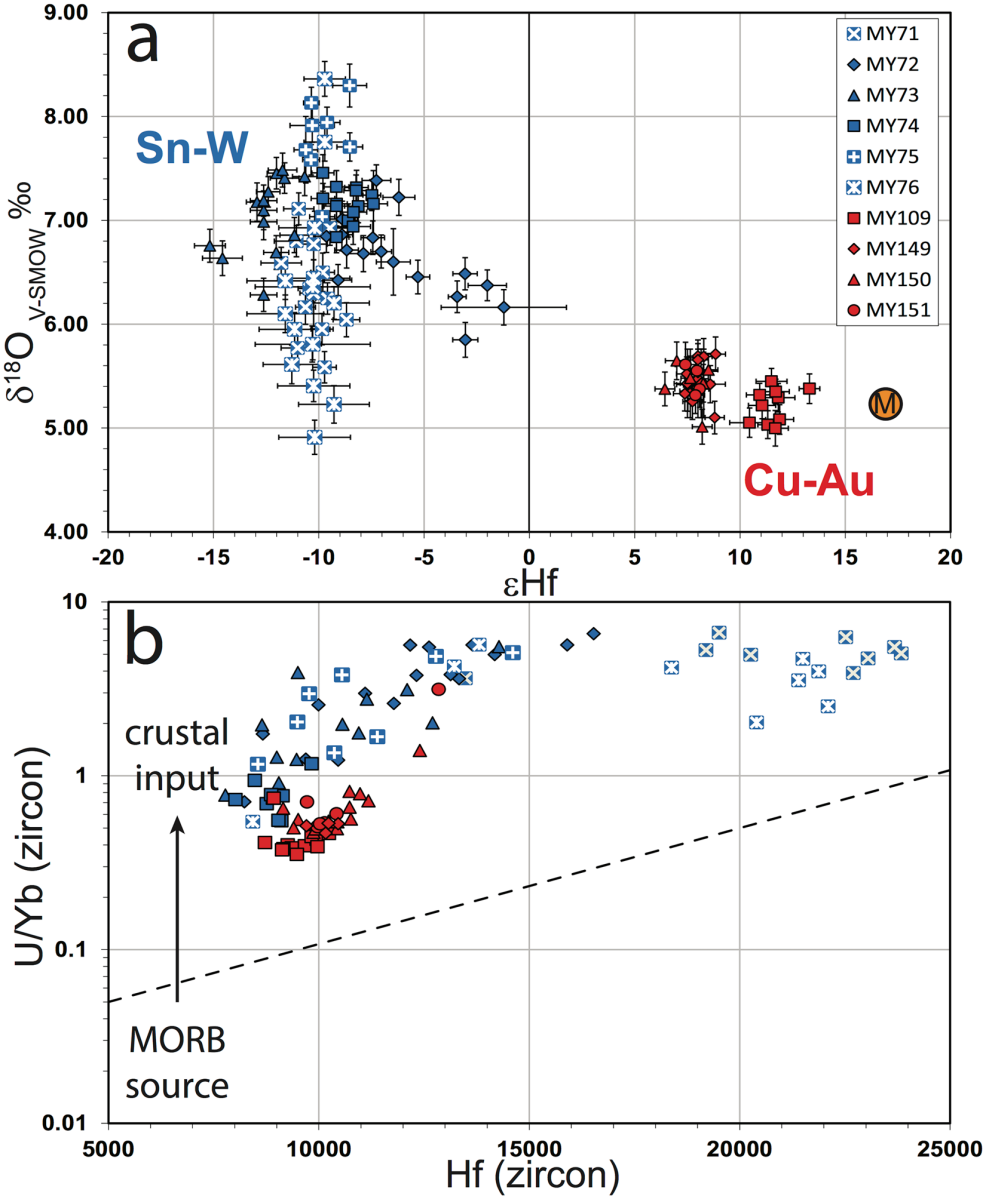
Zircon isotope and trace element plots highlighting source, plotted by sample and suite. Samples MY71, MY75 and MY76 have a high (>15 ppm) Sn content (white crosses). (a) Initial εHf versus δ^18^O; error bars 2σM is a representative mantle value. (**b**) U/Yb versus Hf^5^.

Zircon ^207^Pb-corrected ^238^U/^206^Pb magmatic ages for copper arc granites range from 102-98 Ma, and from 76–58 Ma for the tin belt, consistent with previous studies^16, 17^. Zircons from the copper arc have positive εHf (median values for zircons from different whole rocks range from +7.6 to +11.5) indicating a more juvenile source than those from the tin belt, which have lower εHf implying a more evolved source (median values −7.0 to −13.0). Copper arc samples have a mantle-like median zircon δ^18^O signature of 5.2–5.5‰ V-SMOW. Tin belt rocks have heavier median zircon O isotope values (6.2–7.7‰), also implying greater contributions from pre-existing crust. The δ^18^O-εHf plot (Fig. 2a), therefore, isotopically resolves distinct source compositions for the granitic magmas in the two belts.

Plotting zircon Hf versus U/Yb highlights relative crustal contributions in the parental magmas^5^. Zircons from the tin belt have markedly higher U/Yb ratios than those from the copper arc (Fig. 2b), reaffirming a greater contribution from crustal sources. Two tin belt samples with elevated whole-rock Sn contents (>15 ppm; MY71 and MY76) have higher zircon Hf contents than the other tin belt samples.

Zircon isotopic and trace element signatures reaffirm that the igneous rocks in the two belts have different magmatic ages, are derived from different sources, and they underline the role of geodynamic setting in magma petrogenesis. A juvenile mantle signature dominates the copper arc, and the link between a subducting slab leading to oxidized intermediate magmas and Cu-Au mineralization is well established^18^. Tin belt granites are sourced from isotopically more evolved protoliths with little or no mantle input. Such S-type magmas are typically generated in the mid- to lower-crust through the partial melting of dominantly metasedimentary source rocks, leading to reduced melts that can evolve towards Sn-W mineralization^19^. The interpreted accretionary setting driving the Myanmar magmatism has obvious parallels in both the Central Andes and other magmatic belts originating in similar geodynamic regimes.

### Metallogenic Processes

Blevin *et al*. (1996)^20^ used whole-rock indices for redox (Fe_2_O_3_/FeO) and fractionation (Rb/Sr) to explore the conditions involved in the generation of East Australian ore-bearing granites. They showed certain metallogenic affinities plot within common domains, highlighting the relationship that Cu-Au and Sn-W associated granites have with differing degrees of redox and fractionation. This work provides a useful framework within which to assess the sensitivity of zircon geochemistry to metallogeny.

### Fractionation

**Figure 3:**
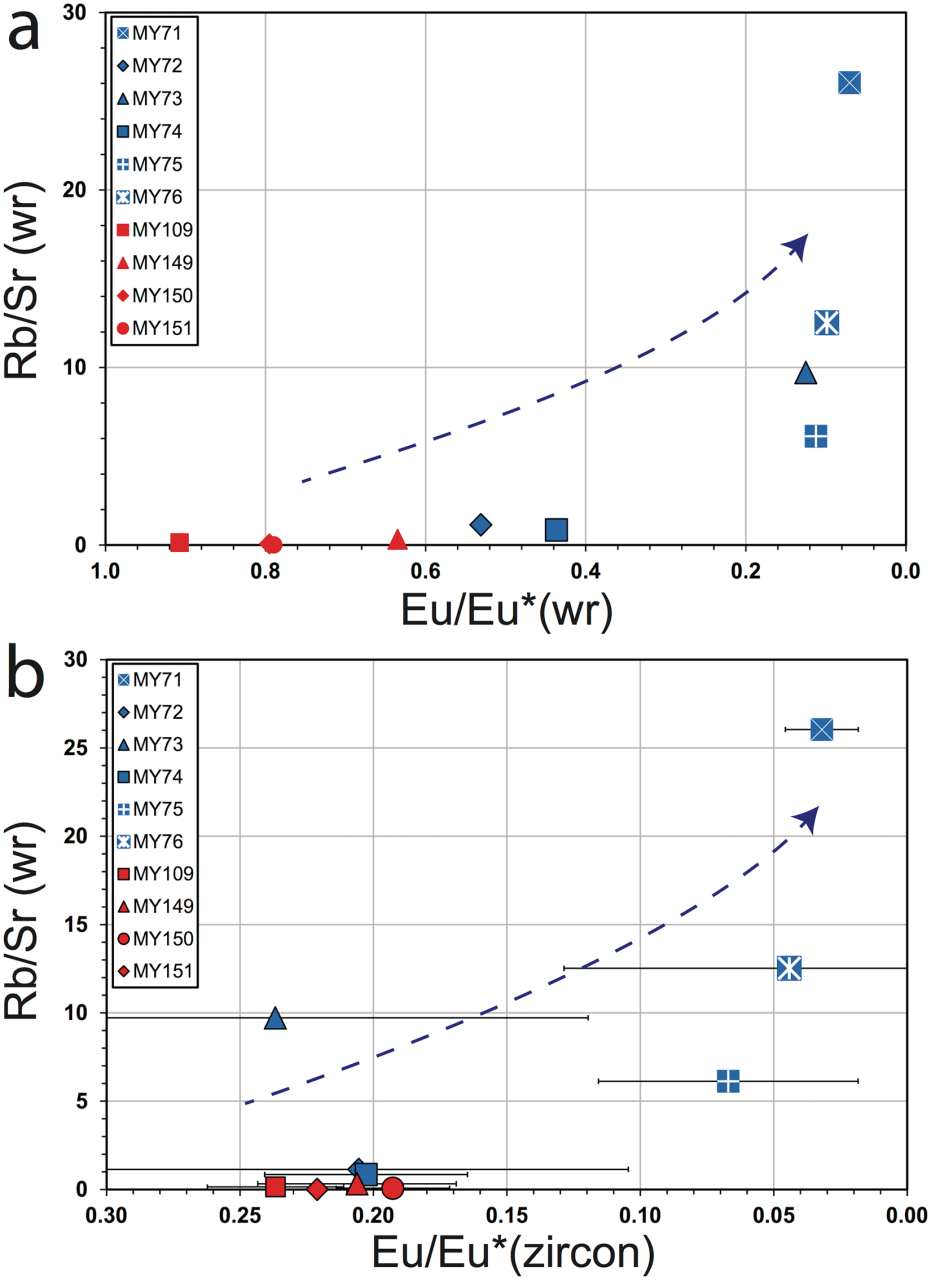
Whole-rock and zircon trace element plots highlighting the relationship between Rb/Sr and Eu/Eu*, plotted by sample and suite. Samples MY71, MY75 and MY76 have a high (>15 ppm) Sn content (white crosses). (**a**) Whole rock Rb/Sr plotted against whole rock Eu/Eu*, and a relationship between these is observed, indicating that Eu/Eu* is sensitive to the degree of magma fractionation. (**b**) Sn belt and Cu arc whole rock Rb/Sr plotted against median zircon Eu/Eu*, showing a relationship that reflects the degree of fractionation of the individual samples. Error bars are 1σ. The zircon Eu/Eu* values are less (i.e. the size of the Eu anomalies are greater) than those in the corresponding whole rock.

Whole-rock Rb/Sr ratios are commonly used to evaluate the degree of magma fractionation. For the Myanmar whole-rock data, the size of the Eu anomaly increases, i.e. Eu/Eu* decreases, with increasing Rb/Sr (Fig. 3a). This Rb/Sr variance is most marked within the tin belt samples; the copper belt samples show limited Rb/Sr variation, although they exhibit a range of whole-rock SiO_2_ (57–77%). Eu^2+^ and Sr are compatible in plagioclase, and so plagioclase crystallization results in residual melts with elevated Rb/Sr and pronounced negative Eu anomalies. Although plagioclase crystallization may be suppressed by the growth of amphibole, reflected in high Sr/Yb ratios^21^, the low whole-rock Sr/Yb values for the Myanmar samples (Sr/Yb < 13) suggests that this is not relevant here. The link between whole-rock Rb/Sr and whole-rock Eu/Eu* is further reflected in the decrease in Eu/Eu* in zircon with increasing whole rock Rb/Sr (Fig. 3b). Thus Eu/Eu* in zircon is an effective monitor of fractionation in evolved magmas, and it appears to be particularly sensitive to fractionation in the Myanmar tin belt samples.

### Redox

Studies of zircons as potential oxy-barometers have focused on the role of the REEs Ce and Eu^8^. Ce^4+^ is significantly more compatible in zircon than Ce^3+^ and, although a function of melt Ce content, systematic Ce/Ce* variation with oxygen fugacity has been demonstrated experimentally^22^. A hindrance to the reliable calculation of zircon Ce/Ce* is the typically low to negligible concentrations of La in zircon, which may be below the level of detection (LOD) for some analytical equipment (e.g., quadrupole LA-ICP-MS), and a potential problem for our copper arc samples. We therefore extrapolated a value for Ce* from Pr and Nd in log scale where La was below the LOD (see supplementary information). Higher Ce/Ce* implies more oxidizing conditions (resulting also in a diminished Eu anomaly). We observe that median values for copper arc zircons have Ce/Ce* ranging from ca. 46–350, while tin belt zircon Ce/Ce* medians are in general less, ranging from ca. 0.8 to 43, and indicating that they were derived from more reducing systems. Our values of zircon Ce/Ce* equate to a range in log *f*O_2_ of between −19 and −34 for the tin belt, and −11 to −18 for the copper arc samples, using the calibration of Trail *et al*. (2012)^23^ for T = 650 °C. These ranges in log *f*O_2_ are consistent with other published figures for Cu-Au and Sn-W deposits^24^.

### Implications for Granite Metallogeny

**Figure 4:**
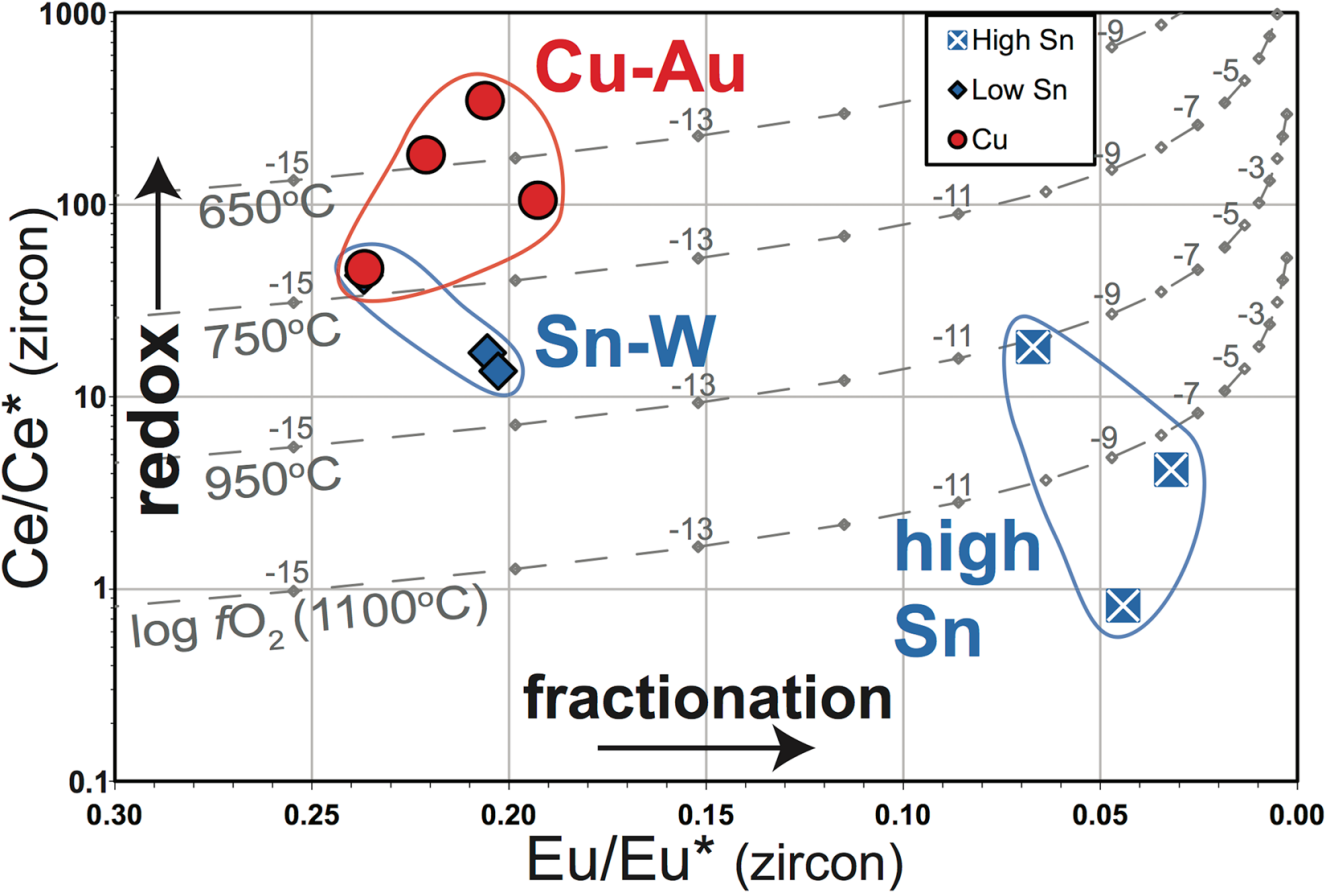
Plot of median zircon Eu/Eu* versus Ce/Ce*. The grey curves represent modelled isothermal covariance of Ce/Ce* with Eu/Eu* as a function of log *f*O_2_ (values annotated) for different temperatures.

Figure 4 plots median zircon Ce/Ce* versus Eu/Eu* for the Myanmar suites. Copper arc zircons define a relatively restricted field with elevated Ce/Ce* and higher Eu/Eu* (more oxidizing, limited range of Eu/Eu*). Tin belt zircons have lower Ce/Ce* and a wider range in Eu/Eu*, and samples with high whole-rock Sn contents notably have significantly lower Eu/Eu – i.e. a greater Eu anomaly - highlighting that they are associated with more fractionated magma compositions (Fig. 3b).

Eu/Eu* is sensitive to both redox and fractionation. To separate these effects we modelled the isothermal co-variance of Eu/Eu* with Ce/Ce* for changes in oxygen fugacity, *f*O_2_, at 650, 750, 950 and 1100 °C, using the calibrations of Trail *et al*. (2012)^23^ for a putative whole-rock composition (ASI 1.25). These plot as trajectories of increasing Ce/Ce* with decreasing Eu/Eu* (Fig. 4). Ce/Ce* is temperature sensitive in these calibrations, and the curves plot at lower Ce/Ce* with increasing temperature. Since S-type tin belt granites have lower Ce/Ce* values and tend to be generated at lower temperatures than the copper arc granites^25^, we conclude that the differences in Ce/Ce* between the zircons of the copper and tin granites reflect differences in oxidation state rather than temperature.

Systems with higher oxidation states will have less Eu^2+^, and hence smaller Eu anomalies, however trends away from the modelled lines towards lower Eu/Eu* reflect sensitivity to magma fractionation, as in the tin belt granites analyzed here. Thus, although Eu/Eu* is sensitive to redox, it may also be used as an index of magma fractionation. The Ce/Ce* versus Eu/Eu* plot for zircons therefore reaffirms the potential of using zircon chemistry to reflect the differences in magma redox and fractionation associated with various magmatic-hydrothermal deposit types, as developed for whole rocks by Blevin and Chappell (1992)^9^.

### Application To Tin Metallogeny

Practical application of zircons as monitors of magma fertility requires calibration of measured proxies. The concentration of Sn in the residual melt increases with magma fractionation until it reaches a critical threshold required for subsequent mineralization processes^26^. Identification of this threshold within zircon would have significant implications for monitoring Sn mineralization potential. At present Sn data in zircon are relatively scarce, so we have sought to identify the degree of magma fractionation, as indicated by Eu/Eu* in zircon, that is associated with elevated Sn contents in the magma, and which are themselves associated with tin mineralization.

**Figure 5:**
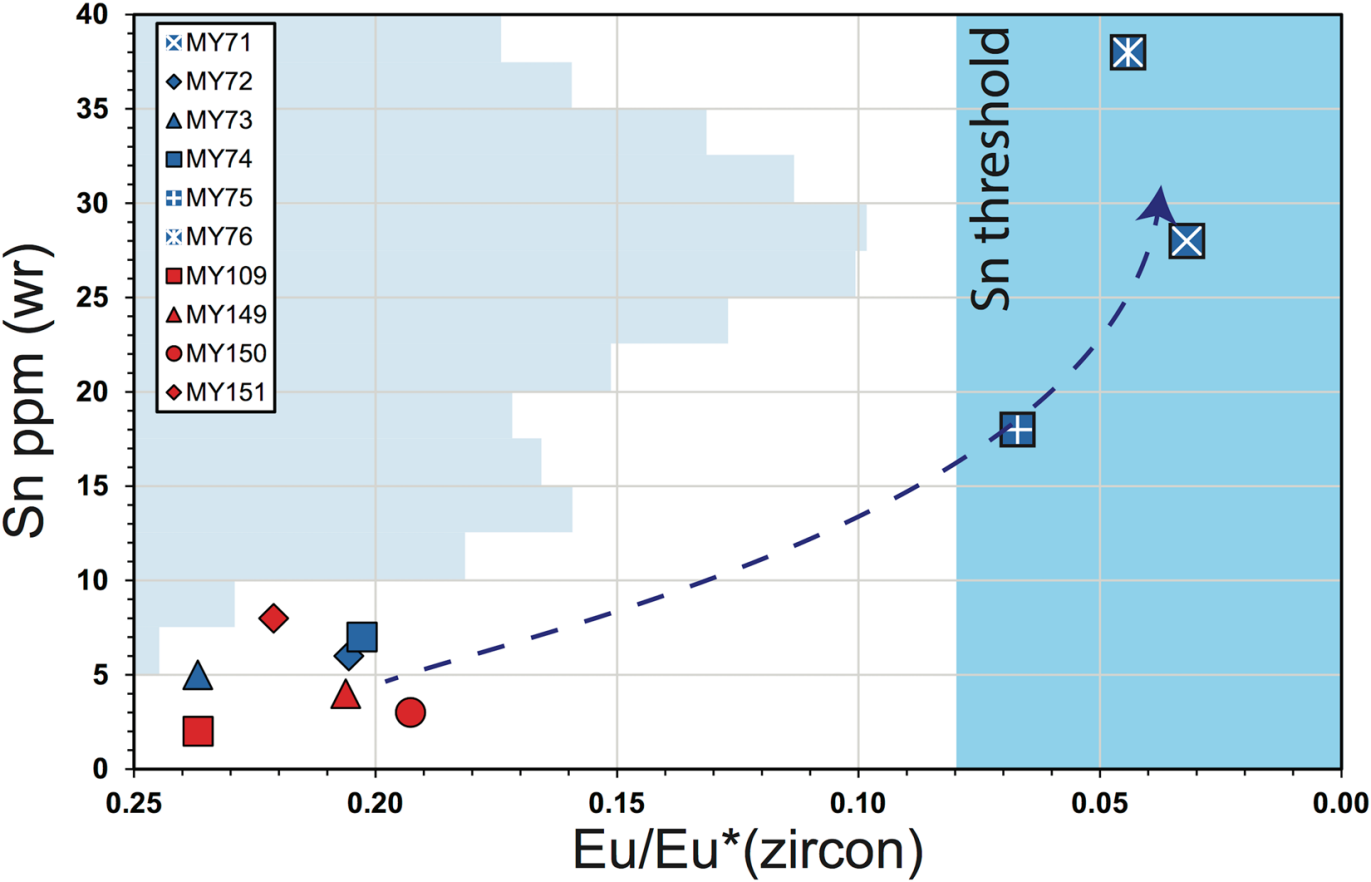
Plot of whole rock Sn content (ppm) against zircon Eu/Eu*. Blue shading represents a “threshold” level of Sn, suggesting a zircon Eu/Eu* of 0.08 or less may indicate elevated Sn values and the potential for the development of tin mineralization. Also plotted on the y-axis are aggregated and bucketed whole-rock Sn contents reported for major tin deposits from Southeast Asia (Malaysia and Thailand), the Erzgebirge, Bolivia, Portugal, Nigeria, and the Massif Central, as summarized in Lehmann (1990)^19^.

Calibration of a Sn threshold can be undertaken with real-world examples. The Zaaiplaats tin deposit is hosted in a granitic phase of the Bushveld Complex that represents a single magmatic pulse. As Zaaiplaats cooled and crystallized, the central residual magma fractionated, concentrating Sn from a background of up to 5 ppm to ca. 35 ppm and promoting cassiterite saturation^26^. Zaaiplaats, however, may be anomalously rich in Sn. Whole rock data from a range of tin-producing granites have minimum values of between 8–25 ppm^19^, as illustrated in the histogram of aggregated whole-rock Sn concentrations in magmas associated with a number of tin deposits from across selected tin provinces^19^ in Fig. 5. Thus, we took a mean of 17 ppm Sn as a target whole-rock Sn content that identifies an economic tin deposit. Plotting whole-rock Sn against zircon Eu/Eu* demonstrates that those samples with elevated whole-rock Sn (MY71, MY75 and MY76) have greater zircon Eu anomalies (Fig. 5). On this plot, a whole-rock value of 17 ppm Sn equates to a zircon Eu/Eu* threshold of approximately ≤0.08.

If Sn in zircon was measured directly, then assessing this threshold in zircon requires knowledge of the partitioning of Sn between zircon and melt. The ionic radius of Sn^2+^ (1.27 Å) is similar to that of Nd^2+^ (1.29 Å) for the zircon VIII coordination site^27^. *Sensu* Blundy and Wood (1994)^28^, the Sn zircon-melt partition coefficient (*D*_*Sn*_) is therefore likely to be similar to that for Nd for more reducing systems. Published values of *D*_*Nd*_ vary, but assuming a zircon-melt value of 3.35^29^, applicable for peraluminous granitoids, a tin mineralization threshold represented by zircon Eu/Eu* ≤ 0.08 may correspond to a zircon Sn value of ≥57 ppm.

## Conclusions

We set out to assess whether source, redox and the degree of magma fractionation may be evaluated from the zircon record of two contrasting magmatic belts. The Myanmar zircon data show that Hf and O isotopes highlight the role of source in determining the initial metallogenic trajectory of resultant magma, with tin belt granites having a more evolved crustal source, comprising material that has previously experienced supracrustal processes, while the copper arc granites have a mantle-like, juvenile, isotopic signature. We set out to recreate the metallogenic affinity diagram of Blevin *et al*. (1996)^20^ through the zircon record, plotting indices of redox versus fractionation. We showed that zircon Eu/Eu* may be a fractionation metric for more evolved magmas. Thus, for the Myanmar rocks, plotting zircon Ce/Ce* versus Eu/Eu* for different suites highlights the differences in redox and degree of fractionation respectively for the Cu-Au versus Sn-W suites (Fig. 4). Further, we specifically see larger zircon Eu/Eu* anomalies – i.e. high degrees of fractionation - for those samples with elevated whole-rock Sn contents.

Work by Ballard *et al*. (2002)^8^ suggested porphyry copper mineralization was associated with magmatic intrusions yielding zircon Ce^4+^/Ce^3+^ > 300 and Eu/Eu* > 0.4. More recently, Lu *et al*. (2016)^6^ suggested that magmatic suites, fertile for Cu-Mo-Au mineralization, in general showed Eu/Eu* > 0.3 and Ce/Nd (their Ce/Ce* proxy) of ca. 2–110. These studies yield smaller Eu/Eu* anomalies (i.e. higher Eu/Eu*) than observed here, inline with what would be expected for less fractionated copper porphyry systems.

Magma fractionation is a key process in the development of enhanced concentrations of incompatible metals, a necessary precursor to mineralization processes leading to deposits of those metals. We use the example of Sn mineralization to show how thresholds may be established in the trace element signatures of zircons, and which may be used to distinguish barren from fertile granites. Zircons have great potential to be developed into a useful exploration tool, especially within the detrital record, and our study suggests they may contain key information regarding pathfinding for lithophile elements.

## Methods

Zircons grains from all samples were separated using a combination of heavy liquid and Frantz magnetic separation techniques. Selected zircons were then mounted in epoxy and imaged using a FEI Quanta 650 FEG Scanning Electron Microscope at the Department of Earth Sciences, University of Oxford, UK. All samples were analyzed for U-Pb geochronology using the large geometry CAMECA IMS1280 ion microprobe at the NordSIM Facility housed at the Swedish Museum of Natural History, Stockholm, Sweden. All calculated ages are ^207^Pb-corrected ^206^Pb/^238^U ages presented at 2σ. Zircon Hf isotopic analyses were conducted with a Thermo Scientific Neptune Plus multi-collector ICP-MS coupled to an ESI New Wave Research 193UC excimer laser ablation system, at the NERC Isotope Geoscience Laboratories (NIGL), Keyworth, UK. Oxygen isotope values in zircon were also measured using the Cameca IMS1280 ion microprobe at NordSIM. Zircon trace element analysis was performed through laser ablation inductively-coupled plasma mass spectrometry (LA-ICP-MS) at John de Laeter Centre, Curtin University, Australia. Zircon grains were ablated using a Resonetics RESOlution M-50A-LR sampling system (incorporating a Compex 102 excimer laser) coupled to an Agilent 7700 ICP-MS. Major and trace element composition of all samples were analyzed at ALS Global Ireland, using ICP-MS (their method ME-MS81d). Full analytical methodology is detailed in the supplementary information, and all analytical results including standard analyses are available in the supplementary data tables in Excel format.

## Electronic supplementary material

Supplementary Information

Dataset 1
