# Supplementary material for: Contrasting Granite Metallogeny through the Zircon Record: A Case Study from Myanmar
Source: Sci Rep. 2017 Apr 7;7:748. doi: 10.1038/s41598-017-00832-2 (PMC5429697; doi:10.1038/s41598-017-00832-2)
Supplement: Supplementary file 1 — Supplementary Information [file 41598_2017_832_MOESM1_ESM.pdf]

Contrasting Granite Metallogeny through the Zircon Record: A Case Study from Myanmar:

Supplementary Information

Nicholas J. Gardiner, Chris J. Hawkesworth, Laurence J. Robb, Martin J. Whitehouse, Nick M.W. Roberts, Christopher L. Kirkland and Noreen J. Evans

Analytical Techniques

U-Pb Geochronology Method

Zircons grains from all samples were separated using a combination of heavy liquid and Frantz magnetic separation techniques. Selected zircons were then mounted in epoxy and imaged using a FEI Quanta 650 FEG Scanning Electron Microscope at the Department of Earth Sciences, University of Oxford.

All samples except MY76 were analyzed using the large geometry CAMECA IMS1280 ion microprobe at the NordSIM Facility housed at the Swedish Museum of Natural History, Stockholm, Sweden, using methods similar to those described by Whitehouse and Kamber (2004)[1] and Whitehouse et al. (1999)[2]. All results used Isoplot for data presentation[3]. All calculated ages are ^{207}Pb-corrected ^{206}Pb/^{238}U ages presented at 2σ. Concordia and weighted average diagrams are presented in Figure S1, and data is provided in the Excel file. Analyses are coded according to the following interpretation scheme: Group I = magmatic; Group D = matrix matching or common Pb issue; Group X = inheritance; Group P = radiogenic Pb loss.

U-Pb Results

The age for most samples is determined from the intersection of a regression line through uncorrected data, which is anchored at the modern day initial-Pb value ($^{207}Pb/^{206}Pb = 0.83$[4]), and the concordia curve. This composition of common Pb has been demonstrated as appropriate for the NordSIM laboratory[5]. This regression approach yields essentially identical results to ^{204}Pb correction of individual ratios, which are also provided in the Excel file. Due to the near concordance of most data, neither the application nor form of common Pb correction results in significant differences to the calculated ages. LA-ICP-MS U-Pb age data for sample MY76 was previously reported in Gardiner et al. (2016)[6].

Sample MY71

15 analyses were performed on 10 grains. Analyses from this sample indicate high to extreme U content that is vastly in excess of that in the 91500 zircon standard used for U/Pb calibration. U content correlates with the 207-corrected $^{238}U/^{206}Pb$ age, in which apparently older dates correlate with higher U content. This pattern implies a matrix-matching issue and hence in preference we use $^{207}Pb/^{206}Pb$ ages. All 15 analyses yield a $^{207}Pb/^{206}Pb$ weighted average of 76 ± 9 Ma (MSWD 2.1), interpreted as the best estimate of magmatic crystallization age of the granite (Fig. S1B, Group I).

Sample MY72

13 analyses were performed on 12 grains. Zircon crystals from MY72 indicate variable U concentrations, and eight analyses points have a U content of >4000 ppm (red; Group D). Those below this have a UO2/U ratio which are within the range of the standard run during the session, hence their U/Pb ratio is regarded as robust. One spot is interpreted to have sampled inherited material and yields an older ^{207}Pb-corrected $^{238}U/^{206}Pb$ age of 73 ± 2 Ma (Spot 02, blue; Group

X). The remaining four analyses when fitted with a regression from modern day common Pb yield an intersection with the concordia curve of 64 ± 1 Ma (MSWD 2.0), interpreted to reflect the time of magmatic crystallization of the granite (Fig. S1C; Group I).

Sample MY73

14 analyses were performed on 8 grains. Seven points from sample MY73 have extreme U contents > 2,000 ppm (red). These analyses are outside the UO2/U range of the standard during the session (Group D). One is interpreted to have samples inherited material and yields an older 207-corrected $^{238}U/^{206}Pb$ age of 61 ± 1 Ma (Spot 06, blue; Group X). The remaining four analyses when fitted with a regression from a modern day common Pb value yield an intersection with the concordia curve of 58 ± 0.5 Ma (MSWD 0.14), interpreted to reflect the time of magmatic crystallization of the granite (Fig. S1D; Group I).

Sample MY74

9 analyses were performed on 8 grains. The analyses are concordant to discordant, principally reflecting a mixture between common and radiogenic Pb. One point shows a significant proportion of common Pb ($f_{207} > 37\%$), and is excluded from further discussion (Group D). The remaining 8 data points (Group I) yield a regression from common Pb which intersects the concordia curve at 59 ± 0.5 Ma (MSWD 0.54), interpreted as the magmatic crystallization age of the granite (Fig. S1E; Group I).

Sample MY75

8 analyses were performed on 5 grains. Three analysis show very high U content >4,000 ppm and are excluded from age calculations due to either or both matrix matching and common Pb contamination issues (red; Group D). The remaining 5 analyses yield a regression from

common Pb which intersects the concordia curve at 72 ± 2 Ma (MSWD 2.8), interpreted as the magmatic crystallization age of the granite (Fig. S1F; Group I).

Sample MY109

8 analyses were performed on 7 grains. Two analysis show younger ^{207}Pb-corrected ^{238}U/^{206}Pb ages, and have high f_{204}%, and are interpreted to have lost radiogenic-Pb (green; Group P). The remaining 6 analyses yield a regression from common Pb which intersects the concordia curve at 102 ± 1 Ma (MSWD 0.44), interpreted as the magmatic crystallization age of the granite (Fig. S1G; Group I).

Sample MY149

17 analyses were performed on 10 grains. The analyses are concordant to discordant, principally reflecting a mixture between common and radiogenic Pb. A regression from recent common Pb was fitted to all analyses, and intersects the concordia curve at 98 ± 0.5 Ma (MSWD 0.88), interpreted as the magmatic crystallization age of the granite (Fig. S1H; Group I).

Sample MY150

8 analyses were performed on 4 grains. Two analyses are interpreted to reflect inherited material and yield older 207-corrected ^{238}U/^{206}Pb ages (blue; Group X). The remaining 6 analyses yield a regression from common Pb which intersects the concordia curve at 99 ± 1 Ma (MSWD 0.38), interpreted as the magmatic crystallization age of the granite (Fig. S1I; Group I).

Sample MY151

4 analyses were performed on a single grain. A regression from common Pb through the data intersects the concordia curve at 98 ± 1 Ma (MSWD 0.05), interpreted as the magmatic crystallization age of the granite (Fig. S1J; Group I).

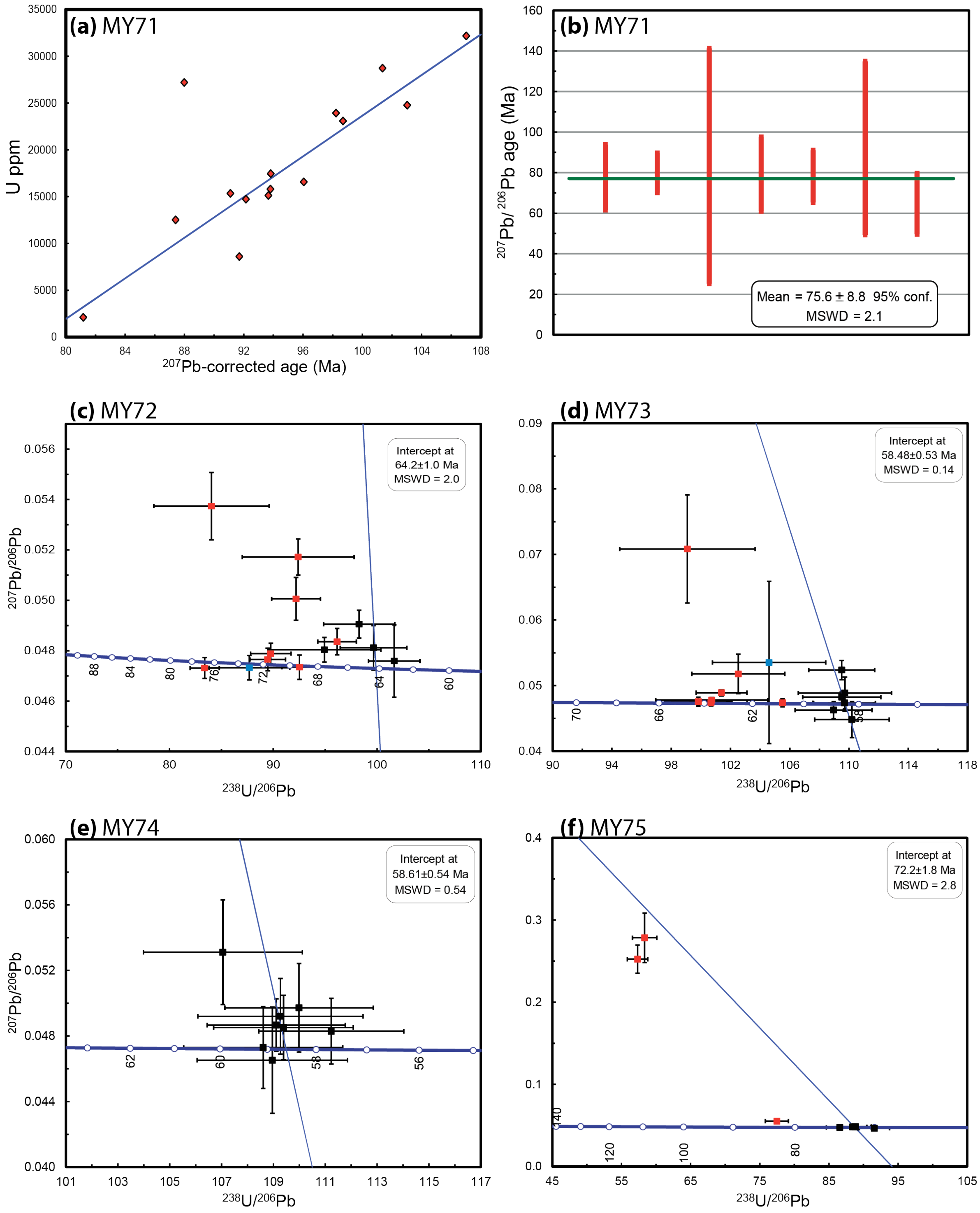

(a) MY71
U ppm
207Pb-corrected age (Ma)
(b) MY71
207Pb/206Pb age (Ma)
Mean = 75.6 ± 8.8 95% conf.
MSWD = 2.1
(c) MY72
Intercept at
64.2±1.0 Ma
MSWD = 2.0
207Pb/206Pb
238U/206Pb
(d) MY73
Intercept at
58.48±0.53 Ma
MSWD = 0.14
238U/206Pb
(e) MY74
Intercept at
58.61±0.54 Ma
MSWD = 0.54
207Pb/206Pb
238U/206Pb
(f) MY75
Intercept at
72.2±1.8 Ma
MSWD = 2.8
238U/206Pb

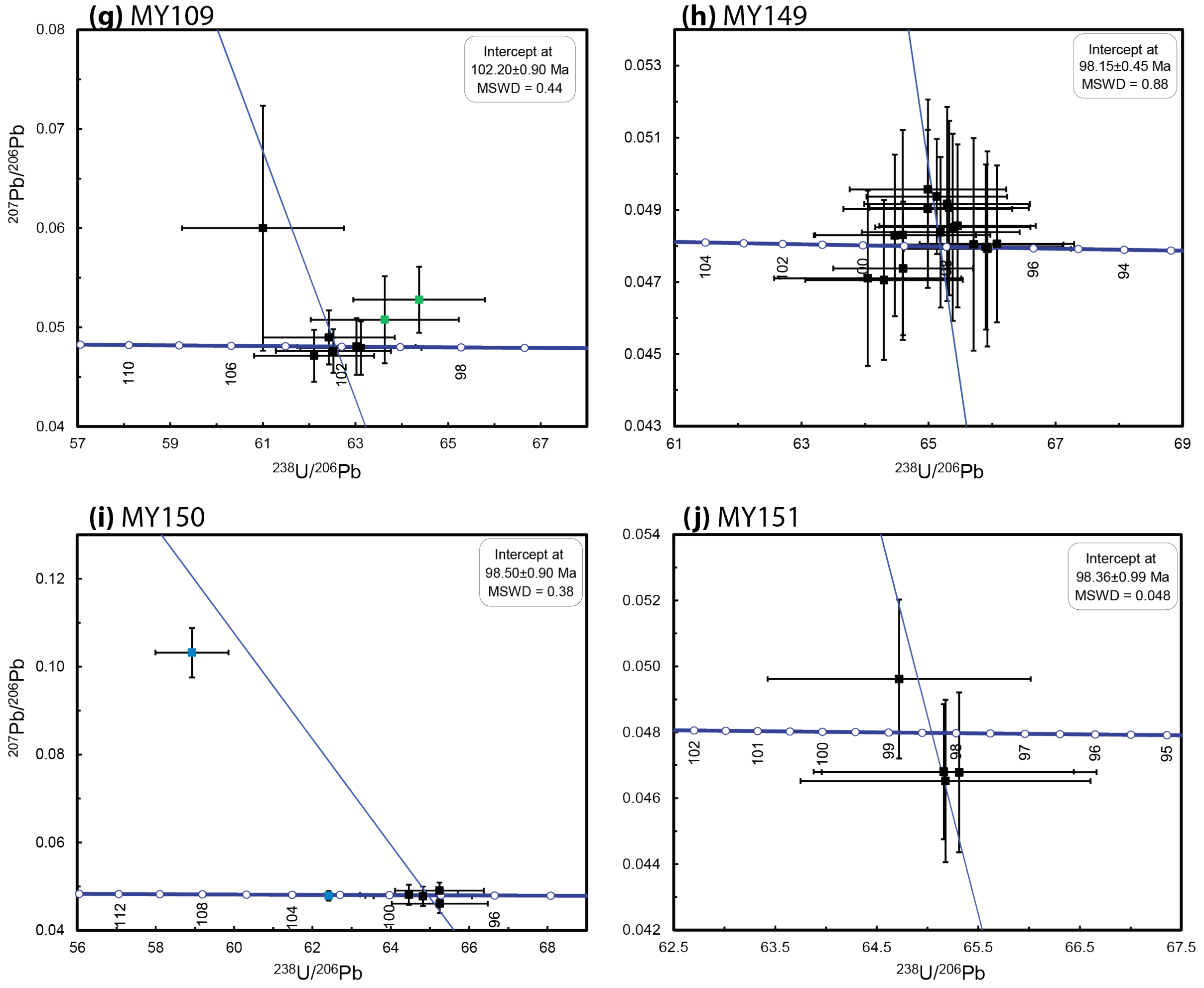

Figure S1: Concordia diagrams showing ^{207}Pb-corrected zircon U-Pb ages, and ^{207}Pb-corrected age weighted average plots, for calculation of Concordia ages. All errors bars are 2σ.

Lu-Hf Isotope Method

Zircon Hf isotopic analyses were conducted with a Thermo Scientific Neptune Plus multi-collector ICP-MS coupled to an ESI New Wave Research 193UC excimer laser ablation system, see Spencer et al. (2015)[7] for the full method description. Helium was used as the carrier gas through the 'TwoVol2' cell, and mixed with Argon at a Y-piece before the torch. Measurement comprised the masses: ^{172}Yb, ^{173}Yb, ^{175}Lu, ^{176}Hf+Yb+Lu, ^{177}Hf, ^{178}Hf, ^{179}Hf and

^{180}Hf, with a 1 second integration time during a 30 second ablation. A 35 μm spot size was used, with a fluence of 7 j.cm^{-2}. Standard sample bracketing using reference material zircons Plešovice, Mud Tank and 91500 [8,9] allowed for the monitoring of precision and accuracy, correction for instrumental drift of the Lu/Hf ratio, and accuracy of the Yb ratio correction. Normalization of the laser ablation Hf isotope data was achieved with solution analyses of reference solution JMC475 (both un-doped and doped with up to 5 ppb Yb). The interference of ^{176}Yb on the ^{176}Hf peak was corrected using a ^{176}Yb/^{173}Yb ratio calibrated for Hf mass bias using the Yb-doped JMC475 solutions[10]. The ^{176}Lu interference on the ^{176}Hf peak was corrected using the measured ^{175}Lu and an assumed natural ratio of ^{176}Lu/^{175}Lu of 0.02653. Systematic uncertainties of Hf and Lu isotope ratios were propagated quadratically, incorporating the external variance of the reference material for each analytical session. The reproducibility of the ^{176}Hf/^{177}Hf ratio of the reference materials ranges from 46 to 56 ppm, and the accuracy based on accepted values is <100 ppm. Iolite 2.5[11] was used for data reduction. Full results of the samples and reference material are provided in the accompanying excel sheet. All uncertainties are reported at 2 sigma. Figure S2 details Hf isotopic data of 91500, Plešovice and Mudtank reference zircons.

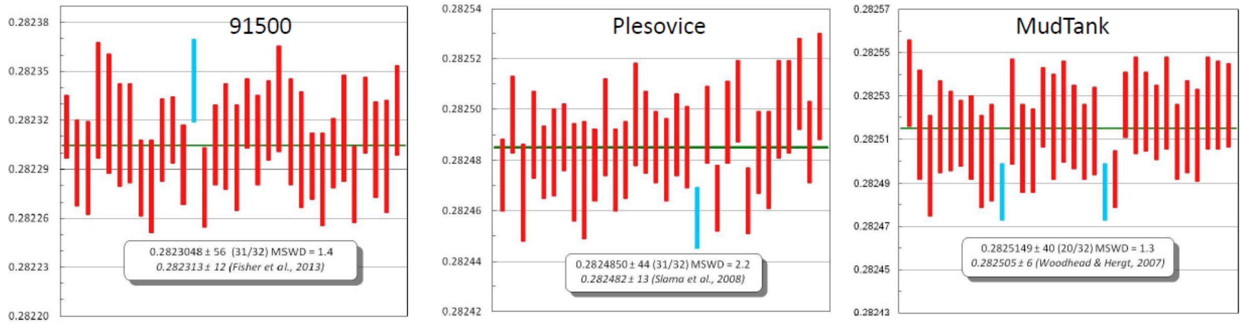

Figure S2: ^{176}Hf/^{177}Hf normalized to JMC475 for zircon reference materials run during the analytical session.

Lu-Hf Isotope Results

Initial $^{176}Hf/^{177}Hf$ ratios were calculated using the decay constant of Scherer et al. (2001)[12]. Figure S3 shows an εHf evolution diagram for the Myanmar samples. Two-stage model ages were calculated assuming a $^{176}Lu/^{177}Hf$ ratio of 0.015[13].

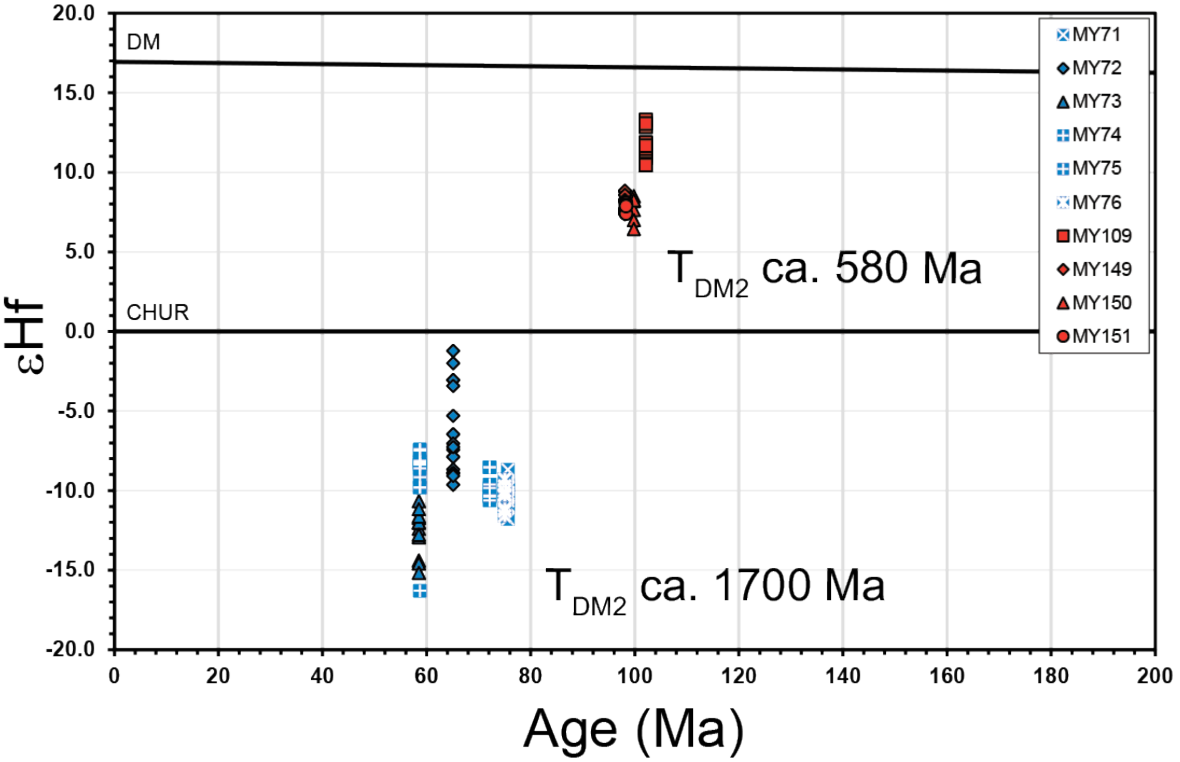

Figure S3. εHf evolution plot for the Myanmar samples.

Oxygen Isotope Method

The oxygen isotope values in zircon were also measured using the Cameca IMS1280 ion microprobe at NordSIM. The analysis was performed with a ca. 2 nA Cs+ primary ion beam together with a normal incidence, low-energy, electron gun for charge compensation, medium field magnification (c. 80×) and two Faraday detectors (channels L′2 and H′2) at a common mass resolution of c. 2500 allowing simultaneous measurement of ^{16}O and ^{18}O. Measurements were

performed in pre-programmed chain-analysis mode with automatic field aperture and entrance slit centering on the ^{16}O signal. The magnetic field was locked using nuclear magnetic resonance regulation for the entire analytical session. Each data-acquisition run comprised a 20 μm× 20 μm pre-sputter to remove the Au layer, followed by the centering steps and 64 s of data integration performed using a non-rastered, c. 10 μm spot. Field aperture centering values were found to be well within those for which no bias has been observed during tests on standard mounts[14]. A total of 82 unknowns were measured during two analysis sessions. In the measurement chain, every set of four unknowns was bracketed by two analyses of the Geostandards 91500 zircon. A $\delta^{18}O$ value of +9.86‰[15] (SMOW) was assumed for the 91500 zircon in data normalization, and small linear-drift corrections were applied to each session. External reproducibility of ±0.11–0.23‰ (1SD), based on the standard measurements, was propagated onto the overall uncertainty for each analysis.

Trace Element Geochemistry Method

Zircon trace element analysis was performed through laser ablation inductively-coupled plasma mass spectrometry (LA-ICP-MS) at John de Laeter Centre, Curtin University. Zircon grains were ablated using a Resonetics RESOlution M-50A-LR sampling system (incorporating a Compex 102 excimer laser) coupled to an Agilent 7700 ICP-MS. Following 15-20 seconds of background analysis, samples were ablated for 30 seconds at a 7 Hz repetition rate using a 33 μm beam spot, and laser energy of 1.5 J/cm^2. The sample cell was flushed by ultrahigh purity He (0.68 L min^{-1}) and N_2 (2.8 mL min^{-1}). Isotopic intensities were measured using an Agilent 7700s quadrupole ICP-MS, with high purity Ar as the plasma gas. In each scan of the mass spectrum, the dwell time for most elements was 0.01 s, with the exception of ^{88}Sr (0.02 s), ^{139}La (0.04 s),

^{141}Pr (0.04 s), ^{204}Pb, ^{206}Pb, ^{207}Pb, ^{208}Pb (0.03 s), ^{232}Th (0.0125 s), and ^{238}U (0.0125 s). International glass standard NIST 610 was used as the primary standard to calculate elemental concentrations other than Hf (using ^{29}Si as the internal standard element and an assumed 14.76 % Si content in zircon) and to correct for instrument drift. Hf in zircon samples was determined using standard zircon GJ-1[16] with ^{90}Zr as the internal standard element. Standard blocks were typically run after 20 unknown analyses. During the time-resolved analysis, contamination resulting from inclusions and compositional zoning was monitored, and only the relevant part of the signal was integrated. The trace element results for NIST 612 (secondary standard) using NIST 610 as the reference material and assuming 33.6 wt % Si, indicate that the accuracy was better than 3% for most elements with the exception of P (5%) and Fe (10%). The analytical precision was better than 10% for most elements.

Trace element data was screened for robustness through a range of techniques, including stability of the time resolved signal (a potential indication of inclusion or mixed domain sampling), and comparing the U-Pb isotope ratios in the trace element dataset with those measured using the smaller volume ion microprobe.

Trace Element Results

Rare earth elements (REE) were normalized to the chondritic values of Palme et al. (2014)[17]. A plot of normalized zircon rare earth element concentrations is shown in Figure S4.

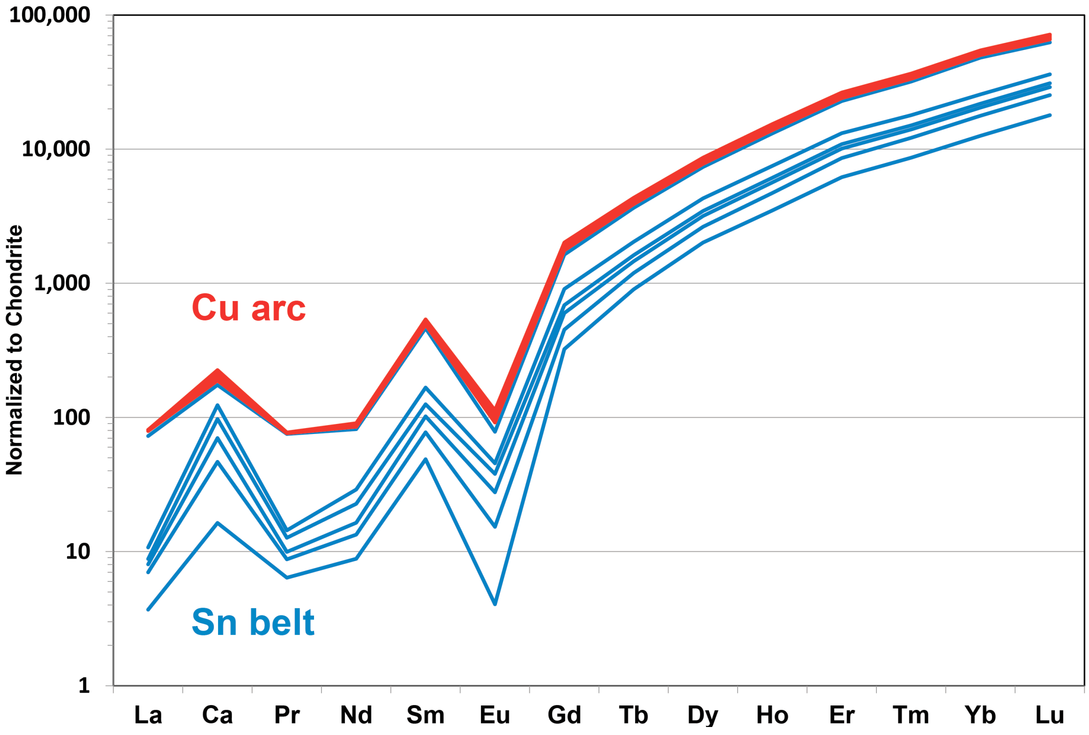

Figure S4. Rare earth element plot for the Myanmar zircons, by belt.

Calculation of Ce and Eu anomalies uses the general forms Ce/Ce* = Ce/(La * Pr)$^{0.5}$ and Eu/Eu* = Eu/(Sm * Gd)$^{0.5}$, using chondrite-normalized values. A hindrance to the calculation of Ce/Ce* is the often low-to negligible concentration of La in many zircons, a particular problem with some of the copper belt analyses. For these samples, we extrapolated a value for Ce* from Pr and Nd in logarithmic scale. Assessment of this approach for analyses where La was measured showed a good fit between Ce/Ce* calculated using a measured value of La, and that calculated using Ce* estimated from logarithmic extrapolation (Fig. S5). Analytical errors were propagated in quadrature both per analysis level, and at sample mean level.

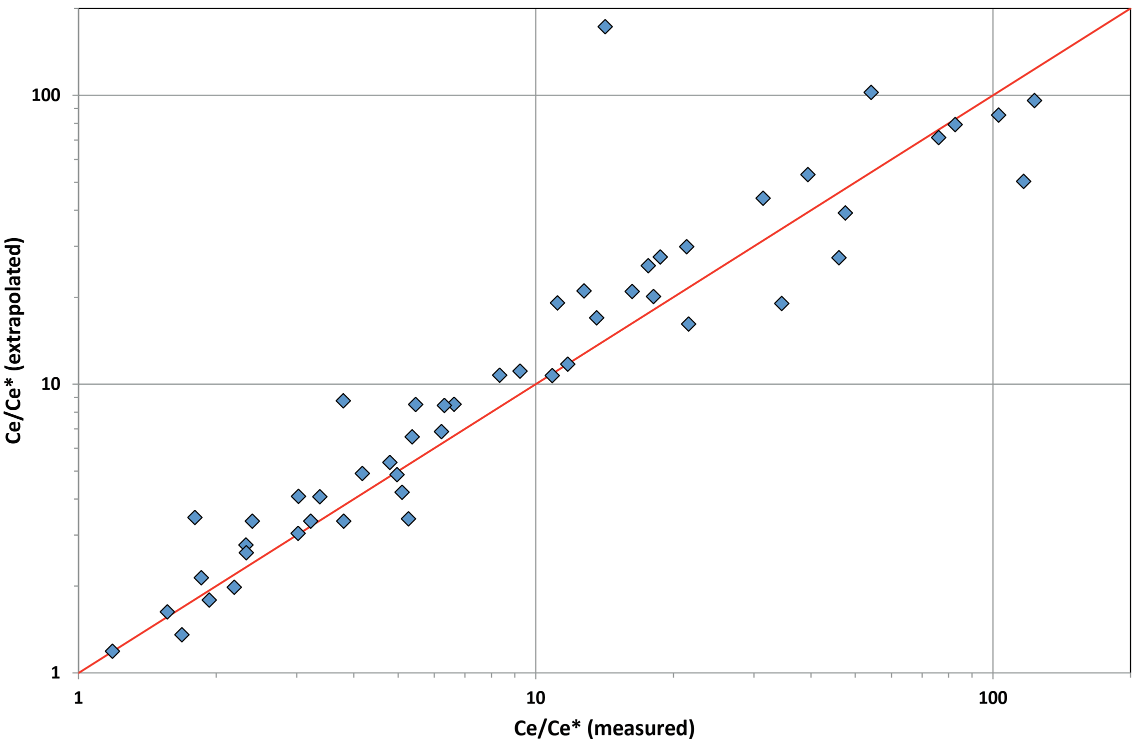

Figure S5. Plotting Ce/Ce* calculated using measured values of La versus Ce* extrapolated from Pr and Nd in log scale.

Bulk Rock Composition

Major and trace element composition of all samples were analyzed at ALS Global Ireland, using ICP-MS (their method ME-MS81d). Samples were prepared through crushing,, milling, and then underwent lithium metaborate fusion. A prepared sample (0.200 g) is added to lithium metaborate ux (0.90 g), mixed well and fused in a furnace at 1000°C. The resulting melt is then cooled and dissolved in 100 mL of 4% HNO_3 / 2% HCl_3 solution. This solution is then analyzed by inductively coupled plasma - mass spectrometry.

Zircon Ce/Ce* versus Eu/Eu*

Plotting Ce/Ce* versus Eu/Eu*, sensitive to redox and fractionation respectively, shows metallogenic affinity for the Myanmar samples in the same sense as the whole-rock (Fe_2O_3/FeO) versus (Rb/Sr) diagram of Blevin et al. (1996)[18]. Figure S6 shows the full dataset plotted. We don't yet fully understand how to interpret the scatter of points so for the purposes of discussion we plot median values per sample.

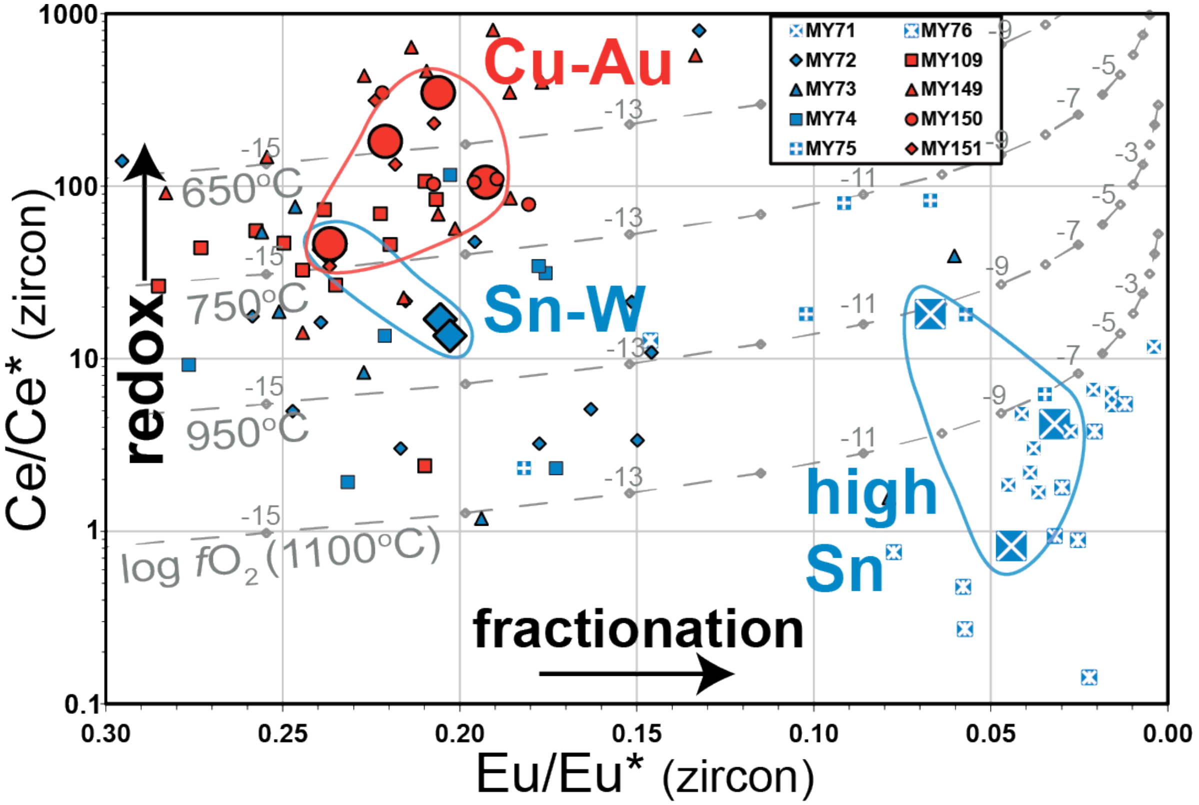

Figure S6. Plot of Ce/Ce* versus Eu/Eu* showing all data points, larger points are median values per sample.

References

1 Whitehouse, M. J. Assigning Dates to Thin Gneissic Veins in High-Grade Metamorphic Terranes: A Cautionary Tale from Akilia, Southwest Greenland. *Journal of Petrology* **46**, 291-318, doi:10.1093/petrology/egh075 (2004).

2 Whitehouse, M. J., Kamber, B. S. & Moorbath, S. Age significance of U–Th–Pb zircon data from early Archaean rocks of west Greenland—a reassessment based on combined ion-microprobe and imaging studies. *Chemical Geology* **160**, 201-224 (1999).
3 Ludwig, K. R. User's manual for Isoplot, 3.16: A Geochronological Toolkit for Microsoft Excel. (2004).
4 Stacey, J. S. & Kramers, J. D. Approximation of terrestrial lead isotope evolution by a two-stage model. *Earth and Planetary Science Letters* **26**, 207-221 (1975).
5 Kirkland, C. L., Daly, J. S. & Whitehouse, M. J. Basement–cover relationships of the Kalak Nappe Complex, Arctic Norwegian Caledonides and constraints on Neoproterozoic terrane assembly in the North Atlantic region. *Precambrian Research* **160**, 245-276, doi:10.1016/j.precamres.2007.07.006 (2008).
6 Gardiner, N. J. *et al.* The tectonic and metallogenic framework of Myanmar: A Tethyan mineral system. *Ore Geology Reviews* **79**, 26-45, doi:10.1016/j.oregeorev.2016.04.024 (2016).
7 Spencer, C. J. *et al.* Generation and preservation of continental crust in the Grenville Orogeny. *Geoscience Frontiers* **6**, 357-372, doi:10.1016/j.gsf.2014.12.001 (2015).
8 Sláma, J. *et al.* Plešovice zircon — A new natural reference material for U–Pb and Hf isotopic microanalysis. *Chemical Geology* **249**, 1-35 (2008).
9 Woodhead, J. D. & Hergt, J. M. A preliminary appraisal of seven natural zircon reference materials for in situ Hf isotope determination. *Geostandards and Geoanalytical Research* **29**, 183-195 (2005).
10 Nowell, G. & Parrish, R. R. Simultaneous acquisition of isotope compositions and parent/daughter ratios by non-isotope dilution-mode Plasma Ionisation Multi-collector Mass Spectrometry (PIMMS). *Special Publication Royal Society of Chemistry* **267**, 298-310 (2001).
11 Paton, C., Hellstrom, J., Paul, B., Woodhead, J. D. & Hergt, J. M. Iolite: Freeware for the visualisation and processing of mass spectrometric data. *Journal of Analytical Atomic Spectrometry* **26**, 2508-2518 (2011).
12 Scherer, E. E., Munker, C. & Mezger, K. Calibration of the Lutetium-Hafnium Clock. *Science* **293**, 683-686 (2001).
13 Griffin, W. L. *et al.* Zircon chemistry and magma mixing, SE China: In-situ analysis of Hf isotopes, Tonglu and Pingtan igneous complexes. *Lithos* **61**, 237-269 (2002).
14 Whitehouse, M. J. & Nemchin, A. High precision, high accuracy measurement of oxygen isotopes in a large lunar zircon by SIMS. *Chemical Geology* **261**, 32-42 (2009).
15 Wiedenbeck, M. *et al.* Further characterisation of the 91500 zircon crystal. *Geostandards and Geoanalytical Research* **28**, 9-39 (2004).
16 Jackson, S. E., Pearson, N. J., Griffin, W. L. & Belousova, E. A. The application of laser ablation-inductively coupled plasma-mass spectrometry to in situ U–Pb zircon geochronology. *Chemical Geology* **211**, 47-69 (2004).
17 Palme, H., Lodders, K. & Jones, A. Solar System Abundances of the Elements. 15-36, doi:10.1016/b978-0-08-095975-7.00118-2 (2014).
18 Blevin, P. L., Chappell, B. W. & Allen, C. M. Intrusive metallogenic provinces in eastern Australia based on granite source and composition. *Transactions of the Royal Society of Edinburgh: Earth Sciences* **87**, 281-290 (1996).
